# Dissatisfaction in Child Welfare and Its Role in Predicting Self-Efficacy and Satisfaction at Work: A Mixed-Method Research

**DOI:** 10.1155/2017/5249619

**Published:** 2017-10-24

**Authors:** Sabrina Berlanda, Monica Pedrazza, Elena Trifiletti, Marta Fraizzoli

**Affiliations:** Department of Human Sciences, University of Verona, Via San Francesco 22, 37129 Verona, Italy

## Abstract

Child welfare organizations are increasingly concerned with challenges emerging from the assessment of social workers' dissatisfaction. This type of service represents the work area where social workers are at greater risk of burnout. Although several studies account for high social workers' burnout scores, they do not systematically dwell upon its sources and roots. In addition, scholars point out that a considerable number of work related issues may be perceived both as a source of dissatisfaction and satisfaction. We assume that there is a need to deepen the understanding of how dissatisfaction's sources may exert an impact on both personal job satisfaction and professional self-efficacy, which are positively associated with well-being at work. The present mixed-method research has two aims: (1) the extensive exploration, applying qualitative methodology, of the perceived sources of dissatisfaction; (2) the attempt to identify the extent to which those sources predict job satisfaction and professional self-efficacy. It is our purpose to further explore which differences emerge by age. The research involved child welfare workers, that is, SWs employed in public child welfare agencies in the North East of Italy. Results show the predominant role of interpersonal trust and mutual respect, as main predictors of both professional self-efficacy and job satisfaction. Practical implications of findings are discussed.

## 1. Introduction

Societal changes are increasingly and severely impacting social workers' (from now on SW) work environment and practices. According to the OECD's (Organization for Economic Cooperation and Development) definition [1], the public sector is the general government sector at the national, regional, and local levels; it accounts for between 20% and 30% of GDP (Gross Domestic Product) in economically developed countries. After the so-called* golden era* [2] of the welfare states, western countries had to cope with two serious economic downturns: the economic crisis of the 1970s and the one which erupted in 2008. Across national boundaries, in the last decade, the weakening of coalitions, which support the welfare state, expenditure retrenchments, and fragmentation of resources, led to institutional, administrative, and welfare programs' changes and thereby to adjustments of service goals and working practices. The increasingly ageing population and the ongoing migration flows are considerably changing the demographic assets of many countries, triggering important shifting of resources' allocation towards new populations in need. In addition, new social risks and needs arise, deteriorating the already troublesome condition of many, also impacting previously unaffected social groups. Moreover, the number of extremely vulnerable single-parent families rose consistently.

Drastic cut in resources compromises sometimes the necessary generational turnover in the public administration and services. In addition, studies in child welfare account for the ageing of the SWs' population in Europe, and Associations of SWs worry about the insufficient following of subsequent generations of workers [3–6].

Social work requires high social competencies and communication skills. This is even more important in child welfare. Since the late 1980s, the ambiguity of the SWs' professional and public role [7] has represented a recurrent and challenging conundrum for SWs. The awkward feeling in facing the users' needs and expectations may trigger unease and what Gibson [8] identifies as “moral distress.” Moreover, scholars [9] argue that the very core of social work lies in the relationship with users. In addition, SWs have to struggle in order to achieve respect and public recognition of their role, despite the informal setting they work in and the sometimes very unstructured and differentiated outcome evaluation procedures.

The lack of* social recognition* and the sense of “not being good enough” [10] make them feel responsible for negative outcomes or even for splitting the children's family up [8, 11]. Defensive strategies are often adopted in facing fear of gaining a bad reputation [12] and shame may arise from the belief that their own abilities, skills, and knowledge are not sufficient to ensure best practices and children's and users' safety and recovery.

Previous studies [13] underline the fact that reflecting on their own professional identity, as well as on theories, values, and devices used in professional practice, can support practitioners dealing with complex work demands. Nevertheless, the complexity of child welfare's service delivery increases when different professionals are all engaged in public service provision. Practitioners have to work collaboratively across disciplinary and methodological boundaries to address the complexity of most problems they have to cope with [14, 15]. SWs, front line workers, health professionals, physicians, nurses, psychologists, teachers, and educational practitioners are interdependent in their task; however, they share responsibility for the service-user's final outcome and they should be able to manage their relationships with ease [16].

Rapidly changing work conditions accelerated the introduction of changes to the service delivery system, and the social pressure experienced by SWs in different domains leads us to underline the need to redirect the scientific attention on how workers perceive and organize each and every potential source of both dissatisfaction and satisfaction. According to scholars [17], the distinct bodies of literature on job satisfaction and burnout in social work are often referred to interchangeably. In fact, according to the literature, a consistent number of both individual and organizational factors [18] are characterized by high ambivalence in SWs' perception: compassionate actions, relationship with users, relationship supervisee/supervisor, and relationship with coworkers and work type.

*Compassionate actions* may be associated with feeling of comfort and relief and not necessarily to stress and emotional exhaustion. The urge to be compassionate and to meet users' need as to act respecting users' self-determination rights often develops in what scientists identify as compassion satisfaction that is in the positive and rewarding feeling about the personal ability to help and support others [19]. Compassion satisfaction may turn into compassion fatigue above all in child welfare, which is recognized to be the service and work area where SWs are at greater risk of burnout [20]. However, research results are controversial. In fact, scholars [18] underline that the type of social service delivered does not have any impact on the level of compassion satisfaction experienced by SWs emphasizing the possible central effect of different variables of individual differences, such as empathy, attachment style [21–23], and the connected abilities to manage interpersonal relationships.

*Relationships with users* may be often fulfilling and rewarding, supporting workers' high intrinsic job satisfaction [24, 25], and working with clients provides often a sense of self-actualization [26]. These relationships may develop into sources of stress and burnout when SWs feel judged or criticized. Above all, in child protection front line service, workers have to cope with users' hostility and sometimes even with aggression. Challenging complex cases could also provide the occasion to recharge energies and enthusiasm for SWs. In addition, working with abused children may represent a source of well-being because of the sense and meaning it gives to work, but it can also contribute to heighten the workers' level of stress and preoccupation because of the frequent negative media exposure and stigmatization [27]. Despite the research based evidence of SWs' high levels of strain and burnout in child welfare services, SWs [28] consider this type of service highly rewarding relating to both intrinsic motivational aspects and perceived social value attributed to interventions for child's safety and protection.

*Supervisors and staff's support* may be of great service to enhance SWs' awareness and coping strategies, but different supervisee may perceive the same supervisor [24, 29] in quite different manners: when behavior is stigmatized and censured unwanted outcomes are possible and the intended support may change into self-criticism and blame.

*The presence of coworkers* and the* relationship *with them may be satisfying and supporting [28]. Trust in coworkers and in other professionals promotes organizational citizenship behavior [30]. Mutual respect experienced in intra- and interservices contexts and interpersonal trust support cooperative behaviors [31] and influence employee satisfaction and as a consequence employee loyalty [32]. Support from coworkers improves job satisfaction [26] and can buffer against the negative effects of work-overload and burnout [24]. Previous studies [33] define social networks and implicit/explicit norms of reciprocity and trustworthiness in the workplace as social capital. Trust and reciprocal support at work enrich and positively affect the subjective work experience. Mutual respect and trust are robustly related to workers' well-being; in fact strong and meaningful interpersonal relationships with coworkers decrease emotional exhaustion fulfilling their need for relatedness. Being misunderstood by coworkers or by other health or educational service staff triggers dissatisfaction and stress [9, 34].

Last but not least,* work type* focused on child protection and their families, lack of resources, uncooperative social environment, and tenuous informal network enhance the worker's strain but can also be perceived as positive challenges, which enable workers to feel satisfied in applying their own skills, experience, and abilities [34, 35] to further develop good practices. Other scholars [24, 36] underline the fact that, in research mainly focused on the so-called “stress industry,” less attention is paid to the positive effects of job features, type of task, and scope. The latter are often sources of job satisfaction contributing to the workers' subjective perception of balance between effort and reward [37]. Not achieving expected outcomes triggers dissatisfaction and a sense of lack of fulfillment, but even the pressure of this value-laden negative experience can be alleviated by achieving a general perspective in social work, recognizing the value and positive SWs' provision of support to the community [38]. The absence of external community support and resources may paralyze SWs; however, it can also be perceived as challenge to accomplish something important and useful.

The above-mentioned, rapidly changing work-contexts in social services and the ambivalence in SWs' perception lead us to accept Zyphur's suggestion [39]. The author invites researchers to apply a more professional-centered or even person-centered approach. The latter gives rise to the need to account systematically for issues and problems professionals perceive as sources of dissatisfaction, rather than to give existing taxonomies for granted. Measuring stress and burnout without analyzing their roots in professionals' perception does not allow us to identify the actual sociopsychological risk factors SWs may run. In addition, it enhances the probability to look for solutions, which engage professionals personally, leaving them alone with their responsibility for health and recovery. Organizational interventions and coping strategies relying exclusively upon professionals' personal engagement in supervision and training activities lead to underestimate the possibility of acting effectively applying a more complex approach based on the interplay of personal, social, ergonomic, contextual, and organizational factors. There is a need to further investigate the perceived sources of dissatisfaction in social work in general and in child protection services in particular and to clarify their relation to professional self-efficacy and job satisfaction, which are typically related to well-being at work.

Self-efficacy (i.e., the sense of competence and mastery) is a critical element for successful coping with stressful conditions (i.e., role and value conflicts) [35], and SWs who score high in self-efficacy are more willing to persevere at work. Professional self-efficacy in social work [40] is known to be positively associated with high performance and job satisfaction [35] and negatively related to burnout and turnover intentions. Previous research [41] found that satisfied SWs are less likely to be plagued by stress symptoms; they are committed to their organization and less likely to quit their job.

Job satisfaction is an emotional reaction to work and it is a function of the perceived relationship between what a SW seeks to gain from his/her job and what the SW perceives his/her job to be offering [42, 43]. SWs' job satisfaction is one of the elements, which can ensure the success of social services in the long-term [44]. The importance of employees' job satisfaction is recognized and its relation with performance, absenteeism, and turnover has been proven in the field of social work [42, 45]. Job satisfaction affects SWs' health, mental health, and social functioning [26]. Previous research [41] found that child welfare workers' satisfaction was predicted by three factors: work, profession, and personal life. In particular, SWs with higher rates of satisfaction have higher overall occupational commitment and organizational citizenship and lower level of intention to turnover and stress related symptoms. Other scholars [42] explored the relationship between SWs' satisfaction and a certain number of factors, which are related on the one hand to personal characteristics (i.e., there is a positive relationship between age and satisfaction) and on the other hand to work conditions (i.e., higher autonomy and lower work-overload), to work rewards, and to work relationships. Highly qualified supervision and satisfying relationships with colleagues are associated with higher levels of satisfaction. Some scholars [34] underlined that the ability and competences to help effectively patients and families, concrete resource provision, job challenge, and autonomy are sources of SWs' satisfaction. Moreover, resources results [33, 46, 47] proved that the way in which people perceive an increase or decrease of workload can become an obstacle to achieving expected outcomes and could compromise the perception of job satisfaction. In fact, the intensity of work affects negatively SWs' job satisfaction and eventually leads to absenteeism and increases workers' turnover intentions; it causes errors, reduces productivity, and increases organizational costs [48]. Work-overload is one of the most important antecedents of burnout [49–51], emotional exhaustion [52–54], and work-life conflict [55–57]. Work-overload plays an important role in the experience of work stress [58, 59], and it is linked with indefinite complaints, fatigue, depression [60, 61], and poor well-being [62]. Work-overload can overtire an individual mentally and physically [63]. However, there are a number of interesting variables and work characteristics, which may tackle these problems effectively: task variety, task significance and meaning, and positive feedback about its impact on others [64–66].

In order to investigate the perceived sources of dissatisfaction in child welfare and to clarify their relation to professional self-efficacy and job satisfaction, we applied a mixed-method research design. We conducted two studies. Study 1 explored what SWs perceive as dissatisfying and uncomfortable at work (i.e., sources of dissatisfaction). Study 2 verified if and to which extent those sources predict professional self-efficacy and job satisfaction.

An additional purpose is to deepen how age influences SWs' self-efficacy and job satisfaction. In fact, previous literature shows that child welfare SWs' age and length of service correlate positively with lower levels of burnout [20]. In addition, younger SWs are more likely than seniors to quit their job [67, 68]. The strength of the relationship between low depersonalization and affective commitment to one's job seems to increase with age and with length of service [69, 70]. Among younger workers high organizational commitment is the unique variable associated with a reduction of emotional exhaustion. Supervisory support [4] makes a difference in safeguarding against stress among younger SWs but not among seniors. Moreover, previous research underlines that young SWs suffer from lack of support and isolation and are often willing to quit their job [4, 71, 72]. According to those results, scholars [4] developed an age-path analysis model in order to better understand the larger number of negative psychosocial outcomes reported by younger SWs [4, 71, 72].

## 2. Method

### 2.1. Ethics Statement

The data for these two studies were collected from two online questionnaires. Ethical approval was obtained from the Ethics Committee at the researchers' institution. Questionnaires included a section that explained the nature and purpose of these studies and a consent form. Informed consent was obtained from each participant, who voluntarily participated in the studies. Participants were informed about their right to withdraw or refuse to give information at any time without incurring any penalties. We protected the privacy and anonymity of answers of individuals involved in our research. The research involved child welfare workers, that is, SWs employed in public child welfare agencies in the North East of Italy.

### 2.2. Study 1: Exploring Risk Factors in Child Protection Services

*Subjects and Data Collection*. SWs reported narratives about sources of dissatisfaction at work. The qualitative analysis was carried out with NVivo 11. The latter allowed us to identify different risk factor categories and to determine relationships and hierarchies among them. This study was carried out between January and February 2017. This study was presented as research on child welfare workers' sources of dissatisfaction at work. We contacted by e-mail 120 SWs employed in public child welfare agencies in the North East of Italy. Their e-mail addresses were collected during a training course held by one of the authors. A total of 73 questionnaires were completed, with response rate of 60.83%. The gender distribution was 3 males (4.1%) and 70 females (95.9%). Most respondents were aged between 20 and 39 years (54.8%), 19.2% were aged between 40 and 49 years, and 26.0% were older than 50 years. About half of the sample (50.7%) had up to 10 years of service, 31.5% had between 11 and 20 years of service, and 17.8% had more than 20 years of service.

*Measurement and Data Analysis*. The questionnaire included the following:

- Three open-ended questions about sources of dissatisfaction at work (“What is your first/second/third source of dissatisfaction at work?”)
- Some questions on demographic and occupational characteristics (gender, age, and length of service)

Qualitative analysis was performed with NVivo 11. We analyzed our data according to grounded theory [73–75] as a methodology. We considered all given and coded answers as subjective indicators of stress. At a different and more complex level of analysis, they were included into indexes of stress. When professionals indicated a source of unease or dissatisfaction, it has to be noted that we captured the subjective perception of the issue and not the issue itself. Each minimum unit of meaning was classified by attributing one or more categories to it [76]. In addition, the explorative study accounts for codes and categories, which can be associated in order to detect related themes and patterns covering an entire semantic area. We gained thereby a detailed overview of the perceived roots of dissatisfaction and of relationships among them. Analysis was structured around two conceptually progressive coding operations (Pedrazza and Berlanda, 2014; Strauss and Corbin, 2008). The first step of the interpretation process was open, also called substantive coding; in this first level of abstraction, the data are explored analytically, fractured, and assembled into superordinate categories (child nodes). In the second step (axial coding), data are organized, summarized, and categories; they are drawn up and grouped into macrocategories (parent nodes); researches therefore investigated the interactions and the links between categories.

We identified four factors of dissatisfaction (parent nodes): lack of trust and mutual respect (49.62%), work-overload (34.21%), employment contractual concerns (8.27%), and difficult cases (7.90%).

**Figure 1:**
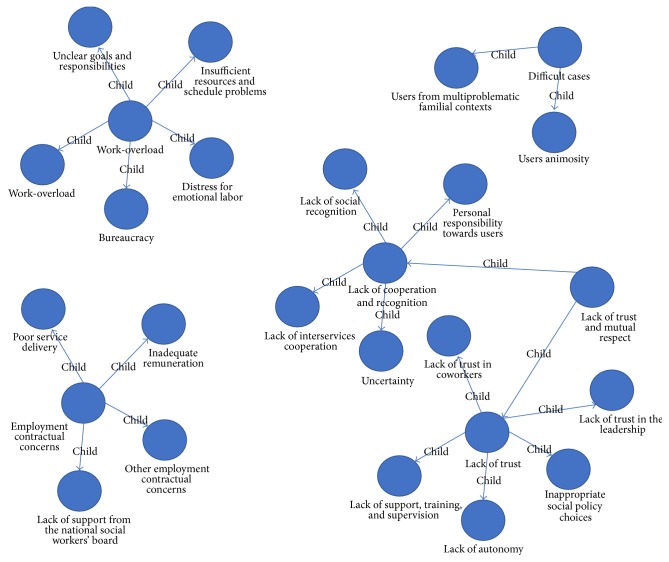
Sources of dissatisfaction: parent and child tree nodes.

As shown in Figure 1, the prevalent source of dissatisfaction at work is lack of trust and mutual respect (parent node). In this area, our participants reported a wide range of issues (child nodes) concerning lack of cooperation and recognition (56.06%, i.e., lack of social recognition, lack of interservice cooperation, uncertainty, and personal responsibility towards users), and lack of trust (43.94%, i.e., lack of trust in coworkers, inappropriate social policy choices, lack of trust in the leadership, lack of support, training and supervision, and lack of autonomy). The second risk factor at work is work-overload (parent node). In this category, there are the following child nodes: work-overload (39.56%), unclear goals and responsibilities (19.78%), insufficient resources and schedule problems (16.48%), bureaucracy (13.19%), and distress for emotional labor (10.99%). The third source of SWs' dissatisfaction at work is employment contractual concerns (parent node). In this area, there are the following child nodes: inadequate remuneration (50.00%), other employment contractual concerns (36.36%), poor service delivery (9.09%), and lack of support from the national SWs' board (4.55%). Finally, the fourth identified risk factor at work is concerned with difficult cases (parent node). This category includes as child nodes users from multiproblematic familial contexts (76.19%) and users animosity (23.81%).

**Figure 2:**
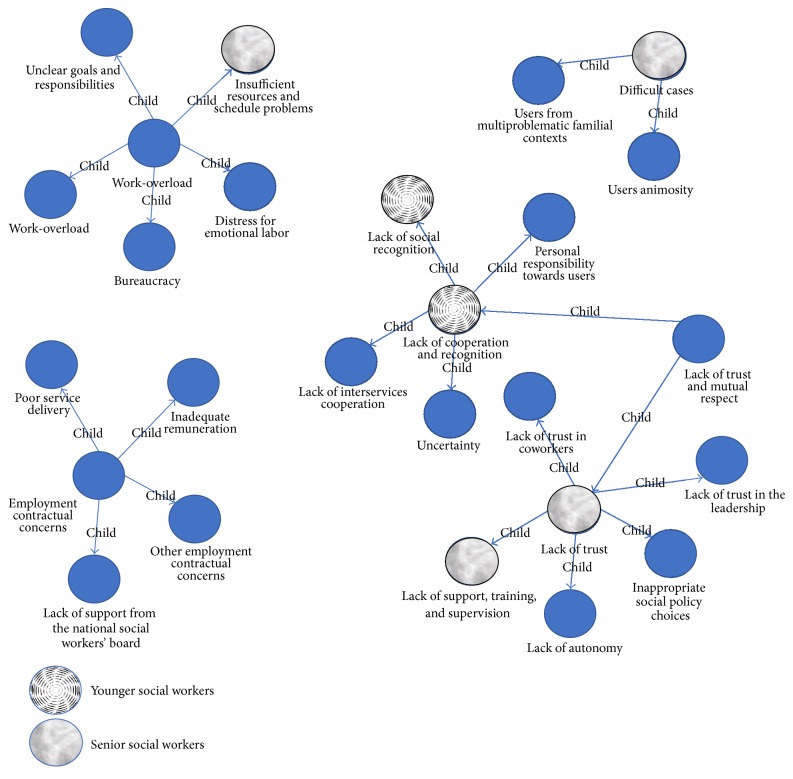
Sources of dissatisfaction's map for* younger* and* senior* social workers' subgroups.

We performed Mann–Whitney tests using SPSS 21.0 in order to explore possible differences regarding the category saturation between senior and younger subgroups (see Figure 2). We divided participants into two groups based on their age. We split the sample into two groups relating to the average of age (from 20 to 39 years and 40 or more of 40 years). Significant differences emerged in lack of cooperation and recognition (*p* < .040) and in lack of social recognition (*p* < .005); these are the major risk factors in the sample of younger SWs. By contrast, senior SWs assigned more importance than younger SWs to lack of trust (*p* < .010), lack of support, training and supervision (*p* < .015), insufficient resources and schedule problems (*p* < .050), and difficult cases (*p* < .015).

### 2.3. Study 2: Lack of Trust and Mutual Respect and Work-Overload Predict Professional Self-Efficacy and Job Satisfaction

*Subjects and Data Collection*. Study 2 investigated the relationship between the two most important identified psychosocial risk factors at work, professional self-efficacy and job satisfaction within a larger sample of child welfare workers. Participants, employed in public child welfare agencies in the North East of Italy, completed a questionnaire including measures of work-overload, trust and mutual respect, self-efficacy, and job satisfaction. This study was carried out on April 2017. This study was presented as research on child welfare workers' well-being at work. We contacted by e-mail 400 SWs employed in child welfare agencies, selected on the basis of voluntary participation.

The sample of study 2 is partly (21.95%) overlapping with the sample of Study 1. Both samples belong to the same geographical area and type of service.

A total of 246 questionnaires were completed, with response rate of 61.50%. The gender distribution was 18 males (7.32%) and 224 females (91.06%); 4 participants have not indicated the gender (1.62%). The mean age was 38.77 years (SD = 9.88; range = 23–60; 2 missing data, 0.81%), and the mean length of service was 12.51 years (SD = 9.35; range = 1–40; 6 missing data, 2.44%).

*Measurement and Data Analysis*. The questionnaire included work-overload, trust and mutual respect, self-efficacy, and job satisfaction. Responses were given on a 7-point Likert scale, ranging from 1* (completely disagree)* to 7* (completely agree)*.

*Work-Overload*. It was evaluated using a four-item measure [77] (e.g., “my workload is heavy on my job”). Cronbach's alphas were .78.

*Trust and Mutual Respect*. In this study, we used six-items: two-items to measure SWs' perceived trust and mutual respect in the relationship with other coworkers (e.g., “I trust my coworkers”), two-items to measure trust and mutual respect in the relationships with different professionals employed in health, educational, and foster services (e.g., “I trust other professionals”), and two-items to measure trust and mutual respect in the relationship with the leadership (e.g., “I trust my leaders”). Cronbach alpha was .86.

*SESSW*. The Self-Efficacy Scale for Social Workers [78] consists of three dimensions: emotional regulation (4 items), procedural self-efficacy (5 items), and support request (3 items). Emotion regulation refers to SWs' confidence in their own ability to manage negative emotions that arise when dealing with complex cases/situations (e.g., “I always manage to keep my anxiety levels within certain levels when dealing with serious situations”). Procedural self-efficacy concerns the ability to deal with different aspects of the social work practice, such as establishing a fair and kind relationship with the user, writing and updating case reports, and not giving up in the face of failure (e.g., “I am always able to fulfill my commitments to the user”). Finally, support request refers to confidence in the ability to look for and find support in other professionals, superiors, and colleagues (e.g., “I am always able to look for and find support from people in other professions”). Cronbach's alpha coefficients for emotion regulation, procedural self-efficacy, and support request subscales were .86, .77, and .86, respectively.

*Job Satisfaction*. In this study, job satisfaction was measured with one item (“I am satisfied with my job”).

Quantitative analysis was performed with SPSS statistical software package; Version 21.0. PRELIS (LISREL 8.7) was used for the imputation of missing data with the expectation-maximization (EM) algorithm, because it provides more accurate estimates of population parameters than list-wise deletion or mean substitution [79]. Only 1.82% of the total responses were missing scores (demographics were not submitted to missing data imputation). First, for each variable, a composite score was computed by averaging the respective items. Pearson correlation was used to examine the association between variables. To test whether younger and senior SWs reported different levels of self-efficacy and job satisfaction, independent *t*-tests were applied. We divided participants into two groups on the basis of their age, using the quartile split method. We split the sample into two groups relating to the average of age (from 23 to 30 years and from 48 to 60 years). Finally, multiple linear regression analyses were conducted: emotional regulation, procedural self-efficacy, support request, and job satisfaction were dependent variables. We included in the regression models work-overload, trust and mutual respect, and age as predictors.

## 3. Results

### 3.1. Descriptive Statistics and Correlations

The means, standard deviations, and correlations of study variables are presented in Table 1. The correlations reveal that trust and mutual respect, professional self-efficacy, and job satisfaction were positively related. Age was positively correlated with the SWs' confidence in their own ability to manage negative emotions that arise when dealing with complex cases/situations.

### 3.2. Multiple Regression Analysis of Variables on Job Satisfaction and Self-Efficacy

The regression analysis with job satisfaction as dependent variable (Table 2), *F*(3,243) = 61.09, *p* < .001, *R*^2^ = .43, shows that trust and mutual respect (*p* < .005) facilitate and support satisfaction at work.

For the emotional regulation self-efficacy (Table 3), *F*(3,243) = 21.27, *p* < .001, *R*^2^ = .21, 21% of the variance in the score were predicted by trust and mutual respect (*p* < .005) and by age (*p* < .005). The regression analysis, with the procedural self-efficacy (Table 4), *F*(3,243) = 19.82, *p* < .001, *R*^2^ = .20, shows that trust and mutual respect on the part of other professionals facilitate this type of self-efficacy (*p* < .005).

Finally, the regression analysis with self-efficacy in requesting support as dependent variable (Table 5), *F*(3,243) = 40.80, *p* < .001, *R*^2^ = .34, shows that trust and mutual respect (*p* < .005) and work-overload (*p* < .020) facilitate and support this type of self-efficacy.

### 3.3. The Role of Age

We divided SWs into two groups on the basis of their age, using the quartile split method. We split the sample into two groups: from 23 to 30 years (*N* = 63) and from 48 to 60 (*N* = 63). One difference emerged: younger subjects (*M* = 4.08; DS = .95) perceived lower self-efficacy in emotion regulation than senior SWs (*M* = 4.59; DS = 1.11; *p* < .005).

## 4. Discussion and Conclusions

Our results are in line with previous studies. Professionals' perceived sources of dissatisfaction have to be considered sociopsychological risk factors, which should be addressed to and measured systematically over time.

The qualitative research phase (Study 1) allowed us to intercept four main categories as sources of dissatisfaction and unease: lack of trust and mutual respect; work-overload; employment contractual concerns; and difficult cases. Relational issues mainly determine SWs' dissatisfaction: lack of trust and mutual respect are the core element and represent the root of dissatisfaction. We could account for differences in subgroups according to their age. Younger SWs' unease is mostly represented by professional isolation and lack of support that is by concerns related to the relational dimension, whereas senior SWs are more concerned with lack of resources, lack of trust, and lack of supporting supervision. In addition, they perceive as consistent source of strain the frequent occasions where they have to struggle with complicated cases. Literature underlines the fact that the resolution of complicated cases in child welfare requires the contribution of professionals working in different services: social, health, and foster services. In fact, SWs have to guide complex decision making processes, which may trigger interprofessional trust issues. While younger workers feel more isolated, senior ones seem to have developed communication skills over time, which enable them to cooperate effectively at the interservice level. Although the latter seem to have overcome the sense of isolation, senior SWs experience trust issues, which arise mainly when individuals are aware of both the need for cooperation and open communication and the associated risks.

Study 2 allowed us to recognize the importance of relational issues within the workplace defined by its different contexts and actors within: the SW-user relationship, the coworkers' cooperation, supervision, and intra- and interprofessional relationships. The relational dimension, defined by subject's rates on trust and mutual respect, exerts the major effects on job satisfaction. Professional self-efficacy is predicted by the quality of the relationships at work and age. If we consider separately the three different dimensions of the Self-Efficacy Scale for Social Workers, we find that the relational dimension affects them all. Moreover, self-efficacy in emotion regulation is also affected by age, whereas self-efficacy in seeking support is also due to work-overload. Interestingly and in line with literature, we can underline that seniors feel more effective than younger SWs in emotion regulation and in general work self-efficacy.

Scholars suggest that this may be due to the fact that seniors are more likely to be committed to their work because of a lack of alternatives, which in turn are available for younger workers and would explain the latter's proclivity to quit. In addition, scholars suggest that senior workers may have developed problem-focused coping skills which are much more effective than emotion-focused coping strategies in ensuring workers' well-being [80, 81]. Problem-focused coping allows SWs to address issues considering both emotional problems and organizational variables. This more comprehensive approach is more effective than a simple focus on emotional and psychodynamic processes.

Moreover, we highlight the significant role of social support and all concerns related to the development and management of relational/interpersonal issues above all among younger workers. The original contribution of this study is represented by the important role SWs attribute to* the relational dimension* (trust and mutual respect) in predicting job satisfaction and self-efficacy. Our findings are in line with previous studies on the role of supervision in social work [82]. Supervision that was experienced as effective, managing conflicts productively, contributed to lower levels of vicarious traumatization. Furthermore, the compliance with the rules of reciprocity among coworkers and the resulting diffuse trustworthiness are commonly related to workers' well-being [33, 83, 84].

Future research could explore the relationship between attachment style and perceived stress in different work related relational domains such as relationship with user, with coworker, with supervisor, and with different professionals who are committed to cooperate in child protection, employed in health, educational, and foster services. In fact, attachment style is recognized to be the variable of individual difference, which determines the way individuals engage in, develop, and manage their interpersonal interactions. In addition, it shapes self-perception and the evaluation of others with specific reference to the value and relevance one accords to relationships in domestic, peer, and work relationships. Finally, different attachment styles are associated with congruent caregiving styles, shaping thereby up-down relationships, the way individuals take care of others, and the extent to which they feel engaged with needy others.

Moreover, it is reasonable to assume that SWs who are provided with sociopsychological training should not experience a great deal of problems in the relational interpersonal domain at work. Furthermore, there is a considerable difference among European countries and worldwide, in terms of training opportunities to acquire relational skills for SWs: in European Northern countries attachment style can be assessed by SWs who are skilled to use this framework in order to assess children's attachment style and parental skills and to provide effective foster placement [85]. In the national context, where the present study was carried out, SWs are not provided with this particular and extremely useful type of training. We argue it could be of interest to social work research and science to analyze within a long-term perspective to which extent educational systems and different training opportunities relate to what professionals perceive as threatening, stressful, or uncomfortable at work.

According to literature [41], there is a need to develop more complex measures of satisfaction and dissatisfaction in child welfare. We assume that a person-centered perspective could contribute to increase the development of interventions supporting workers in order to intercept and enhance workers' organizational citizenship with specific reference to the congruence between worker's goal setting and work environment. A more person-centered perspective focusing also on sociopsychological models, which explain stress as a multifaceted and subjective experience, is in line with the statement [86] that the hallmark of job satisfaction is subjective well-being; workers rely on their own judgment and not on general, theory-based researches' benchmarks. The overwhelming sense of personal responsibility for failure and lack of self-efficacy may develop into an excessive burden above all in beginner SWs' population [4]. We assume that solutions should trigger both professionals' personal engagement in supervision activities and also organizational changes, innovation, and interventions to improve the working conditions according to what, in a given context, workers perceive as detrimental.

We can enumerate some limitation of the present study. Firstly, no causal relationships can be drawn from the findings of our study because of its cross-sectional nature. In addition, our sample consists of a majority of female SWs accounting thereby for the still persisting stereotype that attributes to women the orientation to prefer helping professions, having low need for power, being sensitive to others, and having high needs for affiliation [87]. Finally, no data were collected on organizational variables, environmental work features, and factors except for those indicated by the examined SWs, who were free to choose and indicate each and every source of dissatisfaction they perceived.

## Figures and Tables

**Table 1:** Descriptive statistics and intercorrelations.

	Mean	SD	(1)	(2)	(3)	(4)	(5)	(6)
(1) Age	38.77	9.88	—					
(2) Work-overload	5.02	1.19	−.034	—				
(3) Trust and mutual respect	4.83	1.10	.002	−.109	—			
(4) Job satisfaction	4.78	1.45	.064	−.078	.653^*∗∗∗*^	—		
(5) SESSW emotional regulation	4.38	1.07	.183^*∗∗*^	−.012	.419^*∗∗∗*^	.423^*∗∗∗*^	—	
(6) SESSW procedural self-efficacy	4.60	0.91	.069	.002	.429^*∗∗∗*^	.420^*∗∗∗*^	.516^*∗∗∗*^	—
(7) SESSW support request	4.90	1.16	.076	.056	.561^*∗∗∗*^	.433^*∗∗∗*^	.490^*∗∗*^	.617^*∗∗∗*^

^*∗∗*^ *p* < .01 and ^*∗∗∗*^*p* < .001; SESSW = Self-Efficacy Scale for Social Workers.

**Table 2:** Multiple regression analysis of social workers variables on job satisfaction (*N* = 243).

Variable	*B*	SE	95% CI	*β*	*T*	Sig.
Age	.009	.007	[−0.005, 0.022]	.063	1.291	.209
Work-overload	−.001	.078	[−0.148, 0.159]	−.001	−.015	.988
Trust and mutual respect	.871	.058	[0.748, 0.989]	.655	13.373	.001

*Note*. The 95% bootstrap CIs were computed for unstandardized regression coefficients (1,000 resamples). CI = confidence interval.

**Table 3:** Multiple regression analysis of social workers variables on SESSW emotional regulation (*N* = 243).

Variable	*B*	SE	95% CI	*β*	*T*	Sig.
Age	.020	.006	[0.007, 0.032]	.183	3.185	.003
Work-overload	.032	.052	[−0.070, 0.141]	.036	.623	.539
Trust and mutual respect	.413	.058	[0.293, 0.521]	.423	7.323	.001

*Note*. The 95% bootstrap CIs were computed for unstandardized regression coefficients (1,000 resamples). CI = confidence interval.

**Table 4:** Multiple regression analysis of social workers variables on SESSW procedural self-efficacy (*N* = 243).

Variable	*B*	SE	95% CI	*β*	*T*	Sig.
Age	.006	.005	[−0.004, 0.017]	.070	1.207	.234
Work-overload	.045	.050	[−0.051, 0.148]	.059	1.011	.376
Trust and mutual respect	.368	.058	[0.249, 0.484]	.443	7.617	.001

*Note*. The 95% bootstrap CIs were computed for unstandardized regression coefficients (1,000 resamples). CI = confidence interval.

**Table 5:** Multiple regression analysis of social workers variables on SESSW support request (*N* = 243).

Variable	*B*	SE	95% CI	*β*	*T*	Sig.
Age	.009	.006	[−0.003, 0.022]	.079	1.498	.126
Work-overload	.123	.052	[0.025, 0.228]	.125	2.365	.017
Trust and mutual respect	.615	.058	[0.502, 0.727]	.578	10.918	.001

*Note*. The 95% bootstrap CIs were computed for unstandardized regression coefficients (1,000 resamples). CI = confidence interval.
